# Supplementary material for: Reliability of the retrospective Clinical Interview Schedule Revised (rCIS-R) to assess relapse in depression in primary care patients
Source: PLoS One. 2023 Mar 16;18(3):e0280997. doi: 10.1371/journal.pone.0280997 (PMC10019660; doi:10.1371/journal.pone.0280997)
Supplement: S1 File — (DOCX) [file pone.0280997.s001.docx]

**ANTLER trial rCIS-R: timing questions and five sections used to measure relapse in depression**

**Mandatory questions concerning the last 3 months**

Q1: have you had a spell of feeling sad, miserable or depressed? *(if Yes, the timing and duration questions are asked and follow-up question from section 2)*

Q2: have you been able to enjoy or take an interest in things as much as you usually do? *(If No, the timing questions are asked and follow-up questions from section 1)*

If the answer to Q1 is Yes and to Q2 is No, then the questions from both sections 1 and 2 are asked

If the answer to Q1 is No and to Q2 is Yes, then the sections 1 and 2 are skipped

Example of timing questions asked after mandatory question1:

Timing Q1: how long after our visit did you begin feeling sad, miserable, or depressed

Timing Q2: how many weeks after our last visit did you begin to feel sad, miserable or depressed

Timing Q3: in the past 3 months did you have a period of sadness or depression lasting for 2 weeks or more?

Duration Q: in the last three months did you have a period of low mood, sadness or depression lasting for 2 weeks or more

**Section 1: Depressive mood (during the worst week in the last 3 months)**

Q1: feeling sad, miserable or depressed more than 3 hours in total on any day

Q2: Did you ever become happier when something nice happened, or when you were in company

Q3: lower libido

Q4: restlessness

Q5: doing things more slowly than usual

**Section 2: Depressive ideas or thoughts**

Q1: feeling of guilt

Q2: lower self-esteem

Q3: feeling of hopelessness

Q4: life is not worth living

Q5: suicidal thoughts

**Section 3: Fatigue**

Q1 *mandatory*: noticing getting tired

Q2: cause for feeling tired (*does not count towards depressive symptom score*)

Q3: feeling tired for more than 3 hours in total on any day

Q4: on experience during the worst week

Q5 *mandatory*: lacking in energy

Q6: cause for lacking energy (*does not count towards depressive symptom score*)

Q7: lacking in energy for more than 3 hours in total on any day

Q8: experience during the worst week

**Section 4: Concentration**

Q1 *mandatory*: problems in concentrating on what you were doing

Q2 *mandatory*: problems with forgetting

Q3: degree of concentration on every day tasks

Q4&5: concentration during with worst week

**Section 5: Sleep**

Q1 *mandatory*: problems with trying to get to sleep or with getting back to sleep

Q2: number of nights with sleep problems during worst week

Q3: time trying to return to sleep (*does not count towards depressive symptom score*)

Q4: number of sleepless nights when spent 3 or more hours trying to get to sleep during worst week (*maximum score is two*)

Q5: waking up 2 hours early during worst week

Q6: cause for poor sleep (*does not count towards depressive symptom score*)

Q7 *mandatory*: problems with sleeping more than usual

Q8: number of nights during worst week

Q9: duration of extra sleep during the worst week (*maximum score is two*)

Q10: number of night with 3 hours of extra sleep during the worst week

*Note: the symptom should last for at least 2 weeks or longer in order to count toward relapse*
