# Supplementary material for: Reliability of the retrospective Clinical Interview Schedule Revised (rCIS-R) to assess relapse in depression in primary care patients
Source: PLoS One. 2023 Mar 16;18(3):e0280997. doi: 10.1371/journal.pone.0280997 (PMC10019660; doi:10.1371/journal.pone.0280997)
Supplement: Attachment — Submitted filename: Response_to_reviewers_Dec22.docx [file pone.0280997.s002.docx]

Dear Dr Katrien Janin,

**Re: PONE-D-22-01963**

**Reliability of the retrospective Clinical Interview Schedule Revised (rCIS-R) to assess relapse in depression in primary care patients.**

**PLOS ONE**

Thank you for arranging the second reviewer and for the invitation to submit a revised version of our manuscript. The main aim of our study was to assess the reliability of the rCIS-R. However, we also conducted exploratory analyses of construct validity to enhance our reliability finding and we are grateful to the reviewer for bringing our attention to it. We realised that the term “validity” could be misinterpreted as “external validity”, which is a different approach towards establishing validity. As a result, we clarified that we were addressing construct validity throughout the manuscript improving its quality. We provide a point-by-point response to the editor’s and reviewer’s comments below. The changes are highlighted in the response below.

We have also included:

- A marked-up copy of the manuscript that highlights changes made to the original version. This is uploaded as a separate file labelled 'Revised Manuscript with Track Changes'.
- An unmarked version of our revised paper without tracked changes, uploaded as a separate file labelled 'Manuscript'.

Yours sincerely,

Larisa Duffy

UCL Division of Psychiatry

Maple House, 149 Tottenham Court Road

London W1T 7NF

**Editors’ comments**

PONE-D-22-01963R1

Reliability of the retrospective Clinical Interview Schedule Revised (rCIS-R) to assess relapse in depression in primary care patients.

PLOS ONE

Journal Requirements:
Please review your reference list to ensure that it is complete and correct. If you have cited papers that have been retracted, please include the rationale for doing so in the manuscript text, or remove these references and replace them with relevant current references. Any changes to the reference list should be mentioned in the rebuttal letter that accompanies your revised manuscript. If you need to cite a retracted article, indicate the article’s retracted status in the References list and also include a citation and full reference for the retraction notice.

[Note: HTML markup is below. Please do not edit.]

**Authors response: we have made corrections to the reference list**

Reviewers' comments:

Reviewer's Responses to Questions

**Comments to the Author**

1. If the authors have adequately addressed your comments raised in a previous round of review and you feel that this manuscript is now acceptable for publication, you may indicate that here to bypass the “Comments to the Author” section, enter your conflict of interest statement in the “Confidential to Editor” section, and submit your "Accept" recommendation.

Reviewer #1: All comments have been addressed

Reviewer #2: (No Response)

**Authors’ response: thank you; we are glad to learn that reviewer#1 accepted all our responses to their comments.**

2. Is the manuscript technically sound, and do the data support the conclusions?

The manuscript must describe a technically sound piece of scientific research with data that supports the conclusions. Experiments must have been conducted rigorously, with appropriate controls, replication, and sample sizes. The conclusions must be drawn appropriately based on the data presented.

Reviewer #1: (No Response)

Reviewer #2: Partly

**Authors’ response: we have made a number of changes (listed below) to the manuscript.**

3. Has the statistical analysis been performed appropriately and rigorously?

Reviewer #1: (No Response)

Reviewer #2: Yes

**Authors’ response: thank you**

4. Have the authors made all data underlying the findings in their manuscript fully available?

The [PLOS Data policy](https://eur01.safelinks.protection.outlook.com/?url=http%3A%2F%2Fwww.plosone.org%2Fstatic%2Fpolicies.action%23sharing&data=05%7C01%7Clarisa.duffy%40ucl.ac.uk%7C1c1cac38288649e884d608dacca99537%7C1faf88fea9984c5b93c9210a11d9a5c2%7C0%7C0%7C638047326295404276%7CUnknown%7CTWFpbGZsb3d8eyJWIjoiMC4wLjAwMDAiLCJQIjoiV2luMzIiLCJBTiI6Ik1haWwiLCJXVCI6Mn0%3D%7C2000%7C%7C%7C&sdata=PsVrcsGrr268T1XjGc%2F%2FHFJ3GDW92TWbSRCuZVXEXPs%3D&reserved=0) requires authors to make all data underlying the findings described in their manuscript fully available without restriction, with rare exception (please refer to the Data Availability Statement in the manuscript PDF file). The data should be provided as part of the manuscript or its supporting information, or deposited to a public repository. For example, in addition to summary statistics, the data points behind means, medians and variance measures should be available. If there are restrictions on publicly sharing data—e.g. participant privacy or use of data from a third party—those must be specified.

Reviewer #1: (No Response)

Reviewer #2: Yes

**Authors’ response: thank you**

5. Is the manuscript presented in an intelligible fashion and written in standard English?

PLOS ONE does not copyedit accepted manuscripts, so the language in submitted articles must be clear, correct, and unambiguous. Any typographical or grammatical errors should be corrected at revision, so please note any specific errors here.

Reviewer #1: (No Response)

Reviewer #2: Yes

**Authors’ response: thank you**

6. Review Comments to the Author

Please use the space provided to explain your answers to the questions above. You may also include additional comments for the author, including concerns about dual publication, research ethics, or publication ethics. (Please upload your review as an attachment if it exceeds 20,000 characters)

Reviewer #1: (No Response)

Reviewer #2: Review comments on the manuscript titled "Reliability of the retrospective Clinical Interview Schedule Revised (rCIS-R) to assess relapse in In the study, the authors showed resutls of test-retest reliability of the rCIS-R to assess relapse in primary care patients.

In the study, the authors showed results of test-retest reliability of the rCIS-R as a secondary analysis of an RCT. And additionally, they examined the association between rCIS-R and other self-rated ratings (PHQ-9 and GRQ).

The manuscript has been reviewed by other reviewers and some comments have already been addressed by the authors. To the comments and responses, I have no additional comments.
However, I have other comments as below.

Major comments

My first concern is the lack of a clear demonstration of validity. Reliability is confirmed by the retest method. The validity of the CIS-R, which has already been validated, may have construct validity by using the same questions as the original CIS-R, but it cannot be validated by examining the relationship with the PHQ-9 and the GRQ.
Although the authors declare that they did an exploratory study, it may be an oversimplification to argue that there may be a little validity by adding that exploratory data.

The PHQ-9 measures status over the past 2 weeks, which differs from the rCIS-R in the time period of the measurement. It does not reflect results from the past 12 weeks.
The GRQ is a self-report of a broader disorder that is not indicative of depression, and the duration of the measure differs from the rCIS-R. It may not be sufficient for the external validity of the rCIS-R for the purpose of measuring recurrence of depression.

**Authors’ response: we thank the reviewer for bringing attention to our exploratory analysis of construct validity. We have improved the manuscript and made it clear that our exploratory analysis was designed to assess construct validity. We made it clear now by adding extra test (“to explore construct validity”) on page 6 and (“construct validity”) on page 19. We agree with the reviewer that it would have been theoretically sound to assess the external validity of rCIS- R by administering the original CIS-R, that is already validated measure, on a weekly basis during the 12-week period. However, this method would have been resource intensive and off-putting for participants and we did not have access to such data. Instead of external validity, we examined construct validity. We reasoned that the fact rCIS-R using the same question as a validated measure CIS-R is already a good indicator that our new measure is a valid measure of depression, and we assessed construct validity by choosing three relevant indicators: PHQ-9, GRQ and stopping trial medication. We explain this in strength/limitations section, though we toned down our claims throughout by stressing that it was an exploratory analysis of construct validity.**

The authors may have positioned the validity study as only an exploratory study in the present study. In fact, the title of the paper says "reliability" but not "validity. However, on page 6, line 120, it says "Our aim was to assess the reliability and validity. This should be a cautionary statement because the validity that can be examined with the current method is highly constrained.

**Authors’ response: we have amended the text on page 6, so to stress that it was an exploratory analysis, and it now reads: “Our aim was to assess the reliability and to explore construct validity of the measure used to assess the main outcome”**

In addition, since this study is a secondary analysis incorporated into the original randomized controlled trial, its study design is difficult to understand. Other reviewers may have already noted this point.

In particular, the description of when the rCIS-R was conducted is difficult to understand. Table 1 shows that rCIS-R was performed at 12, 26, 39, or 52 weeks, and most of them were performed at 26 weeks. However, the description in the text does not tell us how this implementation collection was determined. Ideally, it would be better to assign them equally to each timing, or to set a precedent at some point to prevent bias.

**Authors’ response: we have added extra description on page 9 of how we determined the data collection of the repeated measure: “Our intention was for all 478 participants to complete the rCIS-R twice at 26-week follow-up appointment for consistency and simplicity of the fieldwork. However, if participants preferred to complete it at different follow-up appointment, we allowed it. As a result, …”**

Minor comments

It would be good if you could explain somewhere why you decided to evaluate the past 12 weeks and the reasonableness of your decision.

**Authors’ response: we have added the rationale for using the past 12 weeks and added the following text on page 6: “The 12 weeks recall period was chosen because the follow up appointments were roughly spaced at 3 months or 12 week intervals and it was convenient anchor point for participants to remember what has happened since they last met with the researcher. Also, the 12 week interval was considered as appropriate length for participants to remember.”**

As mentioned above, on page 6, line 118, it is not clear whether "The primary outcome" refers to the outcome of the original RCT or this accompanying study to confirm reliability. It is difficult to tell whether "the primary outcome" refers to the original RCT or to this accompanying study to confirm reliability.

**Authors’ response: to differentiate between the original RCT and our study, we have specified “The primary outcome of the main trial…” on page 6**

The description in Table 1 is confusing as to whether it is "mean" or "n".

**Authors’ response: we put “mean” in brackets, so it is easy to differentiate from “n”, Table 1 page 10**

On page 16, line 275, there is the phrase "strong association," but it is unclear on what basis it is described as "strong. The result may not be a very strong association.

**Authors’ response: we have removed the word “strong” on page 16**
